# tRNA and tsRNA: From Heterogeneity to Multifaceted Regulators

**DOI:** 10.3390/biom14101340

**Published:** 2024-10-21

**Authors:** Yun Li, Zongyu Yu, Wenlin Jiang, Xinyi Lyu, Ailian Guo, Xiaorui Sun, Yiting Yang, Yunfang Zhang

**Affiliations:** 1 Clinical and Translational Research Center of Shanghai First Maternity and Infant Hospital, Shanghai Key Laboratory of Signaling and Disease Research, Frontier Science Center for Stem Cell Research, School of Life Sciences and Technology, Tongji University, Shanghai 200092, China; li_yun@tongji.edu.cn (Y.L.); yuzy@tongji.edu.cn (Z.Y.); jiangwenlin@tongji.edu.cn (W.J.); luna_lvxinyi@icloud.com (X.L.); guoailian@shphschool.com (A.G.); sunxiaorui2007@gmail.com (X.S.); 2 NHC Key Laboratory of Reproduction Regulation, Shanghai-MOST Key Laboratory of Health and Disease Genomics, Shanghai Institute for Biomedical and Pharmaceutical Technologies (SIBPT), Shanghai 200032, China

**Keywords:** tRNA heterogeneity, tRNA modifications, tsRNA, epigenetic regulation, translational control

## Abstract

As the most ancient RNA, transfer RNAs (tRNAs) play a more complex role than their constitutive function as amino acid transporters in the protein synthesis process. The transcription and maturation of tRNA in cells are subject to stringent regulation, resulting in the formation of tissue- and cell-specific tRNA pools with variations in tRNA overall abundance, composition, modification, and charging levels. The heterogeneity of tRNA pools contributes to facilitating the formation of histocyte-specific protein expression patterns and is involved in diverse biological processes. Moreover, tRNAs can be recognized by various RNase under physiological and pathological conditions to generate tRNA-derived small RNAs (tsRNAs) and serve as small regulatory RNAs in various biological processes. Here, we summarize these recent insights into the heterogeneity of tRNA and highlight the advances in the regulation of tRNA function and tsRNA biogenesis by tRNA modifications. We synthesize diverse mechanisms of tRNA and tsRNA in embryonic development, cell fate determination, and epigenetic inheritance regulation. We also discuss the potential clinical applications based on the new knowledge of tRNA and tsRNA as diagnostic and prognostic biomarkers and new therapeutic strategies for multiple diseases.

## 1. Introduction

The traditional view holds that transfer RNAs (tRNAs) are universally expressed housekeeping molecules, stably expressed and involved in cellular protein synthesis processes. However, the expanding evidence supports that cellular, mature tRNA pools, similar to the transcriptome, exhibit strong tissue specificity and cell type specificity, which further brings to light the concept of “tRNA heterogeneity” to characterize their diversity [1,2,3,4]. The heterogeneity of tRNA pools encompasses the variation of cellular overall tRNA abundance, the relative abundance of specific tRNA compositions, amino acid charging levels, and the diversity of RNA modifications. In fact, tRNAs possess the greatest number, diversity, and prevalence of various types of RNA modifications [5]. On average, approximately one-fifth of the nucleotides in tRNA sequences are modified [6]. These chemical modifications of tRNAs are of significant importance for mature tRNA folding and stabilization, anticodon–codon interaction affinity, and decoding fidelity [7,8,9]. Beyond tRNA modifications, the regulation of tRNA aminoacylation levels mediated by aminoacyl–tRNA synthetases is also particularly important for tRNA functions in cellular translation control [10,11]. The tRNA codon landscape and tRNA charging sufficiency directly control cellular translation efficiency during various biological processes [12,13], such as environment stress response [14], stem cell fate determination [15,16], tissue development [17], and cancer progression [18,19,20,21].

Additionally, it has been suggested that tRNAs can function beyond translation control. Some tRNAs could serve as primers for reverse transcription transposons [22] or bind to DNA to hinder the transcription of tRNA genes [23]. Investigating this could expand our understanding of tRNA regulatory mechanisms. Moreover, it is fascinating that tRNAs not only function as intact molecules but could also be reborn from fragmentation [24]. The generation of tRNA-derived small RNAs (tsRNAs) from mature tRNAs revealed a brand new identity and utility for fragmented tRNAs. As a class of non-canonical regulatory small RNAs, tsRNAs could not only undergo the direct regulation of gene expression on transcriptional, post-transcriptional, and translational levels but also engage in crosstalk with other epigenetic factors, such as DNA methylation and histone modifications, to enrich the information compacity and regulatory dimensions of tRNA/tsRNA code [25,26].

With the advancement of high throughput sequencing technology, growing evidence has unveiled distinctive tRNA pools and tsRNA signatures between cancer samples and normal tissue [27,28], revealing the diagnostic and prognostic potential of tRNA and tsRNA profiles in various diseases. Moreover, due to the genetic decoding capacity of tRNA molecules, it is promising to engineer tRNAs to develop new therapeutic strategies for diseases induced by genetic nonsense mutation [29,30]. Based on the increasing body of literature about tRNA and tsRNA, here, we aim to provide an up-to-date summary of the recent advances in our understanding of tRNA and tsRNA, highlight their regulatory mechanisms in various biological processes and diseases, and discuss their potential therapeutic applications based on the new knowledge of tRNA and tsRNA.

## 2. The Heterogeneity of the Mature tRNA Pool

**Figure 1:**
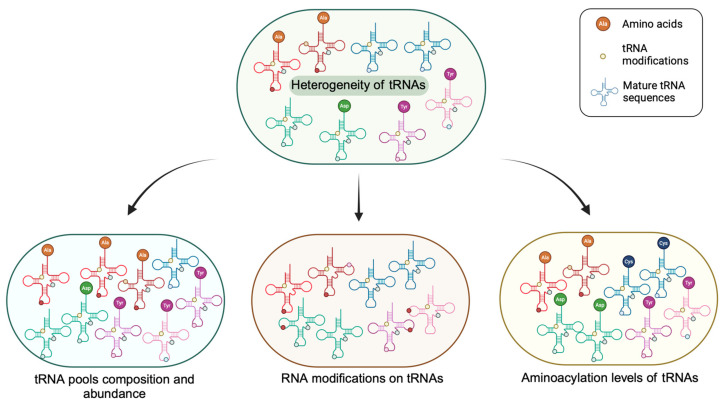
Transfer RNA (tRNA) heterogeneity is manifested in multiple aspects. tRNA heterogeneity is reflected in overall tRNA abundance; the composition of tRNA isotypes, isoacceptors, and isodecoders; the abundance of various RNA modifications; and the aminoacylation levels in cellular tRNA pools, which may be regulated by multiple effectors, such as chromatin accessibility, transcription factor binding, and RNA polymerase III (Pol III) activity, as well as the abundance and activity of tRNA-modifying enzymes and aminoacyl-tRNA synthetases (AARS). The tRNA isotypes are distinguished by different color schemes, and the varying shades within the same color range in-dicate different tRNA isoacceptors. The different colored dots on the tRNA sequence represent different types of RNA modifications. tRNAs are not always fully aminoacylated and that the level varies depending on cell type and growth conditions. Created in BioRender. Yang, Y. (2024) BioRen-der.com/a28u622 (accessed on 15 August 2024).

Traditional tRNA research has primarily focused on the physiological and biochemical functions of specific types of tRNA molecules or tRNA-related enzymes (such as modification enzymes and aminoacyl–tRNA synthetases). However, advances in high-throughput omics technologies have revealed a comprehensive profile of tRNA, encompassing the total abundance of tRNA pools, the composition of specific tRNAs within these pools, the charging levels of tRNAs, and their chemical modifications (see Section 3). These features of cellular tRNA pools have been found to exhibit unique patterns across different tissues and cell types (Figure 1). Such unique patterns of tRNA pools, namely, tRNA heterogeneity, operate globally to control downstream protein translation and potentially play roles in tissue-specific differentiation, stem cell fate determination, and the development and progression of diseases [31,32].

### 2.1. Total Abundance Variation

Protein synthesis in tissues relies on the participation of sufficient amounts of tRNAs. Due to variations in metabolic levels and energy demands across different tissues, there is also variability in the demand for tRNA abundance. Initially, Pan Tao and colleagues [31] primarily revealed this correlation using tRNA microarrays to quantify the total tRNA abundance across eight different tissues and discovered several-fold differences. Next-generation sequencing technologies further allow investigators to quantify the proportion of tRNAs to total RNA in various tissue cells by adding Spike-in controls. The regulation of tRNA abundance is crucial for the adaptation of cellular metabolic patterns, which is primarily governed by RNA polymerase III (Pol III) and its regulatory factors [33].

### 2.2. Composition Ratio Difference

tRNAs are highly diverse and can be classified into isotypes, isoacceptors, and isodecoders based on specific criteria: isotypes differ in the amino acids they carry, isoacceptors have variations in their anticodon sequences in the same isotype, and isodecoders show sequence differences in the same isoacceptor. Studies have shown that the dwell time of ribosomes at the A site is strongly negatively correlated with the abundance of tRNA isoacceptors [34], illustrating how the abundance of specific tRNA isoacceptors impacts the translation of specific codons. This effect can lead to the regulation of translation rates for messenger RNAs (mRNAs) enriched with particular types of codons. Studies employing next-generation sequencing of tRNAs have revealed significant differences in tRNA expression profiles between different tissues and cancer subtypes [27,35,36]. These variations may be associated with tissue-specific development, the maintenance of high translation rates for certain genes, or the severity of cancer [27,31,36]. These findings suggest that the composition of tRNA pools could serve as a regulatory node for selective protein translation.

### 2.3. Charging Level Sufficiency

tRNAs require aminoacyl–tRNA synthetases to attach amino acids to their 3′ end to function effectively. The amino acid charging levels directly affect the availability of tRNAs for peptide chain synthesis, thereby impacting translation processes [37]. Researchers have identified variations in tRNA charging levels across different tissues. For instance, MinXin Guan and others utilized acid urea polyacrylamide gel electrophoresis (PAGE) techniques to examine 15 types of aminoacylated and non-aminoacylated mitochondrial tRNAs (mt-tRNAs) in mouse brains, hearts, livers, skeletal muscles, and kidneys, revealing distinct differences in mt-tRNA charging levels across these tissues [38]. Next-generation sequencing has also been employed to measure tRNA charging levels. For example, mim-tRNAseq (modification-induced misincorporation tRNA sequencing) takes advantage of β-elimination and oxidation processes to distinguish between charged tRNAs, which end in CCA, and uncharged tRNAs, which end in CC [4]. This method was applied in *Trm7*Δ *S. cerevisiae* to discover that specific tRNA species, such as tRNA^Phe(GAA)^, experienced a significant reduction in charging levels compared to the wild type [4]. These uncharged tRNAs lack the capacity to contribute amino acids during peptide elongation but could play roles in the non-canonical functions of tRNAs (such as being primers or binding to DNA and proteins, see Section 4.1) or lead to the formation of tsRNAs.

## 3. Chemical Modifications: Guardians of tRNA Integrity and Function

### 3.1. RNA Modification on tRNA

**Figure 2:**
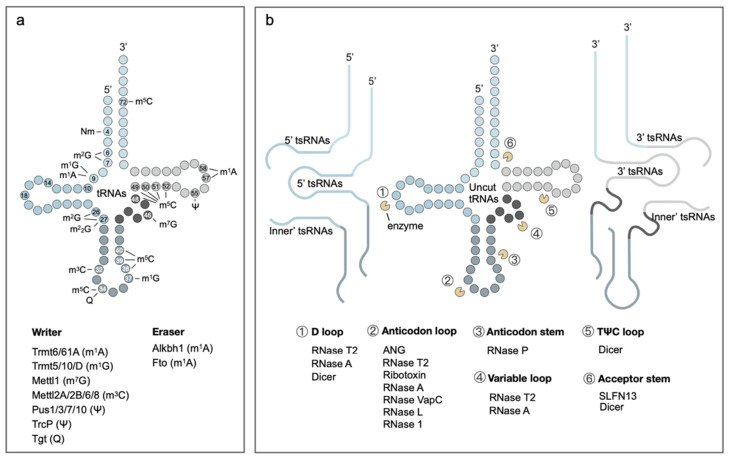
RNA modification on tRNAs and biogenesis of tsRNAs. (**a**) Possible tRNA modification types, sites, and corresponding enzymes. (**b**) 5′tsRNAs generate from D loop nuclease or anticodon loop nuclease; 3′tsRNAs generate from TΨC loop or anticodon loop nuclease; inner’tsRNAs generate from D loop and TΨC loop nuclease.

tRNAs contain the highest diversity and abundance of RNA modifications, with an average of 13 modifications per eukaryotic tRNA molecule, accounting for approximately one-fifth of its nucleotides [5] (Figure 2a). These modifications are catalyzed by specific modification enzymes, which could enhance tRNA stability and translation efficiency, maintaining overall cellular homeostasis, and play crucial roles in various biological processes [39,40,41]. Due to the great variety of tRNA modifications, a complete exposition is beyond the scope of this article. We highlight seven exemplary tRNA modifications that are prevalent and representative. It should be noted that some other tRNA modifications are also crucial, such as taurine methylation modifications (e.g., τm5U and τm5s2U) present in mitochondria, and highly correlated with mitochondrial diseases such as MELAS (mitochondrial encephalomyopathy, lactic acidosis, and stroke-like episodes) [42] and MERRF (myoclonic epilepsy with ragged-red fibers) [43].

#### 3.1.1. 5-Methylcytidine (m^5^C)

m^5^C modifications are distributed on multiple positions of tRNA and modified by diverse modification enzymes, such as NOP2/Sun RNA methyltransferase 2/3/4/6 (NSUN2/3/4/6) [44,45,46] and tRNA (cytosine(38)-C(5))-methyltransferase TRDMT1/DNMT2 [47]. Additionally, alpha-ketoglutarate-dependent dioxygenase homolog 1 (ALKBH1) was reported to further oxidize m^5^C to 5-formylcytidine (f^5^C), while Ten-eleven translocation methylcytosine dioxygenase 2 (TET2) oxidizes m^5^C to 5-hydroxymethylcytidine (hm^5^C) [48,49]. Evidence has shown that m^5^C can protect tRNAs from fragmentation by nucleases. For example, DNMT2 could methylate C38 to m^5^C38 on tRNA^Asp^, tRNA^Val^, tRNA^Glu^, tRNA^Gln^, and tRNA^Gly^, which further stabilized these tRNAs from cleavage at the anticodon site [47,50,51]. The genetic deletion of *Dnmt2* in mouse testis and sperm facilitates the biogenesis of tsRNAs [52]. In addition to DNMT2, the NSUN family shows a broader methylation range of tRNA m^5^C, of which NSUN2 is responsible for the generation of m^5^C at positions 48, 49, and 50 of tRNA [53], while NSUN6 is responsible for the methylation of C72 of tRNA^Thr^ and tRNA^Cys^ [46,54]. NSUN3 is responsible for the initiation generation of m^5^C at the wobble position of mt-tRNA^Met t^ [55], which is further oxidized by ALKBH1 to generate 5fC [56]. The mt-tRNA^Met^ with 5fC34 is capable of reading AUA codons as methionine in mitochondria. The existence of NSUN3 and ALKBH1 is pivotal for cells to enhance the translation rate via 5fC34 mt-tRNA^Met^ to maintain the function of mitochondria. Moreover, the aberrance of other NSUN family proteins can trigger a variety of downstream dysregulations due to the absence of m^5^C modification on tRNAs. Notably, the deletion of *NSun2* in testis destabilizes tRNAs and reduces the levels of several tRNAs, which further impairs spermatogenesis by modulating the translation of RNA processing proteins [57]. In addition, the deficiency of NSUN2 in neurons significantly decreases the abundance of tRNA^Gly^ and subsequently suppresses the translation of Gly-rich proteins, which ultimately leads to impaired synaptic signaling [51]. A summary of m^5^C and other tRNA modifications related to pathologies is shown in Table 1.

In light of the evidence for the demethylation of tRNA m^5^C, Tet family proteins were identified as being responsible for the oxidization of m^5^C on tRNAs. Particularly, TET2 showed a strong affinity with tRNA m^5^C, especially tRNA^Gly^, and could oxidize tRNA m^5^C to hm^5^C both in vivo and in vitro. The deletion of *Tet2* in mouse embryonic stem cells (mESCs) significantly reduced the abundance of tRNA hm^5^C, while overexpression of the catalytic domain of TET2 increased the abundance of tRNA hm^5^C, with an obvious decrease in tRNA m^5^C [48], suggesting a direct functional role of TET2 on tRNA.

#### 3.1.2. N1-Methyladenosine (m^1^A)

The m^1^A modification on tRNAs occurs at positions m^1^A9, m^1^A14, m^1^A22, m^1^A57, and m^1^A58 of different tRNAs across many life forms from bacteria to mammals [58,59]. Among them, m^1^A58 is the most universal methylation position that occurs on tRNAs across all three kingdoms of life, while the other positions of m^1^A are relatively rare. Due to the chemical characteristic of m^1^A, m^1^A could block the Watson–Crick base pairing and play an important role in maintaining tRNA structural stability and folding correction. A lack of m^1^A9 and/or m^1^A58 reduces the thermostability of tRNA L-shaped tertiarily structures and induces the cleavage of tRNAs to generate tsRNAs. In addition, m^1^A is a reversible nucleotide chemical modification, which can be “written” and “erased” by its corresponding enzymes. The tRNA Methyltransferase6/61 (Trm6/Trm61) serves as the writer of m^1^A58 modifications [60], while ALKBH1/2/3/7 and fat mass and obesity-associated proteins (FTO) function as the erasers of m^1^A. The deletion of m^1^A writer Trmt61A in thymocyte induces a global decrease in tRNA m^1^A58 modification abundance and impairs the translation efficiency of Myc mRNA, preventing the proliferation of T cells following antigen stimulation [61,62]. The TRMT6/61A complex also shows dimethylation capacity that methylates tRNA A58 into m^1,6^A58.

ALKBH1 and ALKBH2/3 were first reported to function as DNA repair proteins; however, increasing evidence has shown that they can serve as erasers of tRNA m^1^A modification. For instance, ALKBH1 was reported to demethylate tRNA m^1^A inside cells and tune translation initiation and elongation by modulating the cellular abundance of tRNA^iMet^ and other m^1^A-modified tRNAs that enter the translation machine [63]. ALKBH2/3 were reported to not only show significant m^1^A demethylation capacity on various tRNAs but also on mRNA and CAG repeat RNA. Moreover, ALKBH2/3 can also demethylate m^3^C on tRNA, mRNA, and CAG repeat RNA [64,65,66,67]. High levels of ALKBH3 in cancer cells decrease the abundance of m^1^A and m^3^C on tRNAs and promote cancer progression by facilitating the biogenesis of tsRNAs [68]. ALKBH7 was recently found to be capable of demethylating m^1^A and m^2^_2_G of several pre-tRNAs in mitochondria and functioning as a regulator in mitochondria polycistronic RNA processing. Knockout of ALKBH7 impairs mitochondrial protein translation and suppresses mitochondrial activity by reducing the abundance of steady-state mitochondria-encoded tRNA within m^1^A and m^2^_2_G modifications [69].

Another m^1^A demethylase, FTO, was first discovered as an m^6^A eraser [70] and later confirmed to be able to demethylate m^1^A in tRNAs. Cross-Linking and Immunoprecipitation High Throughput Sequencing (CLIP-Seq) showed a strong binding affinity between FTO and tRNAs. Knockdown of FTO significantly increased the abundance of cellular m^1^A in tRNAs, while the overexpression of FTO led to a decrease in tRNA m^1^A levels [71]. Interestingly, the researchers also investigated the recognition strategy of FTO to distinguish different RNA species as demethylation substrates, especially for tRNAs. The results showed that FTO preferentially binds to stem-loop RNAs and shares a similar overall structure with NSUN6, which could bind to tRNA to methylate m^5^C72, thus promoting the demethylation efficiency of FTO to tRNA m^1^A58 [72].

#### 3.1.3. N1-Methylguanosine (m^1^G)

m^1^G modification usually occurs on position G9 of the D loop and G37 of the anticodon stem-loop of tRNA [73]. In yeast, m^1^G9 was methylated by Trm10, a S-adenosyl-L-methionine-dependent tRNA methyltransferase [74], while m^1^G 37 was methylated by TrmD in *Escherichia coli* (*E. coli*) and TRM5 in eukaryotes and archaea [75]. Interestingly, primary m^1^G methylation at the anticodon position of 37 in tRNA^Pro^ is necessary for adding the modification on wobble position U34, which is of great importance to maintain reading-frame accuracy during translation, suggesting a hierarchical ranking of different RNA modifications on tRNA. m^1^G37 itself also plays a key function in preventing ribosomal frameshift errors and enhancing translation efficiency and fidelity during protein synthesis procedures [76,77]. The mutation of *TRMT5* in humans abolishes its function to methylate tRNA at G37 and causes a series of diseases, such as hereditary spastic paraparesis [78], neuropathy syndrome [79], combined oxidative phosphorylation deficiency 26 (COXPD26) [80], idiopathic non-cirrhotic portal hypertension, and hepatopulmonary syndrome [81], etc., which are associated with the impaired translation efficiency and fidelity in mitochondrial proteins that depend on the correct methylation of mt-tRNA m^1^G37 by TRMT5. Instead, the upregulation of TRMT5 in human cells is associated with tumor progression. Although the regulatory mechanism is not clear, it is reported that TRMT5 was increased in hepatocellular carcinoma (HCC) and correlated with poor prognoses. This might be associated with TRMT5-mediated enhanced tRNA function in translational control [82].

Notably, besides the mutation of TRMT5 affecting the m^1^G methylation levels of tRNA, mutations on tRNA genes also have important roles in regulating tRNA function in translation. For instance, the m.4295A>G mutation of tRNA^IIe^ introduces the base alteration of positions 37A to 37G and methylates to m^1^G 37 on tRNA^IIe^ by TRMT5, which further affects the structure and function of tRNA^IIe^ to impair mitochondrial function and leads to a deafness phenotype [83]. Another example is the m.4435A>G mutation of mitochondrial DNA, which introduces an m^1^G 37 modification into tRNA^Met^. The incorporation of m^1^G 37 in tRNA^Met^ increases the melting temperature and electrophoretic mobility of tRNA^Met^ while decreasing the aminoacylation and steady-state levels of tRNA^Met^, consequently affecting the translation efficiency of mitochondrial proteins [84]. These studies highlight the importance of appropriate tRNA modification levels, as neither hypomodification nor hypermodification on tRNA could maintain the biological function of tRNA.

#### 3.1.4. N7-Methylguanosine (m^7^G)

The modification of m^7^G is one of the most prevalent RNA modifications located at the 5′cap of mRNA, internal ribosomal RNA (rRNA), and tRNA across all species, and recent studies have identified its new location on internal mRNA. The mRNA cap m^7^G is modified by RNA guanine-N7 methyltransferase in complex with RNMT-activating miniprotein (RNMT/RAM); responsible for promoting mRNA processing, export, and translation initiation; and protects mRNA from degradation by various nuclease, while m^7^G on internal mRNA modified by methyltransferase-like 1/WD repeat-containing protein 4 (METTL1/WDR4) is reported to function as a cellular translation regulator. METTL1/WDR4 is also responsible for the methylation of positions 46G to m^7^G46 at the variable loop of about 22 different types of tRNA [85,86,87,88]. The METTL1-WDR4 complex recognizes the elbow of target tRNA through shape complementarity, while the N-terminal of METTL1 coupled with the WDR4 C-terminus and tRNA variable loops to form a catalytic pocket to secure the methyltransferase activity of METTL1 [89,90].

Growing evidence has unveiled the important role of tRNA m^7^G46 in tRNA metabolism. The deletion of METTL1 significantly reduces the m^7^G levels of both tRNA and mRNA and impairs overall translation efficiency in cells [91]. In fact, the loss of tRNA m^7^G46 increases tRNA sensitivity to RNase A/T in vitro and promotes the biogenesis of tsRNA in vivo, which subsequently disturbs the cellular steady-state tRNA pools and inhibits overall protein synthesis. Moreover, the downregulation of m^7^G46 on some tRNAs, such as tRNA^Arg^, can induce ribosome pausing during translation and decoding processes, thus impairing the cellular translation bias that adapts to a high proliferation rate of tumor cells.

In light of the methylation activity of the METTL1-WDR4 complex, increasing evidence confirms the oncogenic role in various cancers, such as acute myeloid leukemia [86,92], bladder cancer [93], hepatocellular carcinoma [94], prostate cancer [95], etc. The expression levels of the METTL1-WDR4 complex are prevalently higher in tumor samples and associated with poor prognoses in patients [86,96]. The deletion of METTL1 reduces cancer cell proliferation and promotes cell apoptosis, thus inhibiting cancer metastasis. Moreover, METTL1-WDR4 complex-dependent tRNA m^7^G46 plays pivotal roles in stem cell fate determination and aging [16,97]. Knockout of *Mettl1* alters cellular tRNA expression signature and leads to self-renewal- and neural differentiation-defective phenotypes in mESCs [16].

#### 3.1.5. N3-Methylcytidine (m^3^C)

On human tRNA, m^3^C usually occurs at various positions such as C32, C20, and C47:3 on cytosol tRNA (cyto-tRNA), while only one position of m^3^C32 was identified on mt-tRNA [98]. The methylation of m^3^C on human cyto-tRNA^Thr^ and cyto-tRNA^Arg^ is catalyzed by METTL2A/METTL2B, while METTL6 catalyzes m^3^C32 on human cyto-tRNA^Ser^ [99]. However, the methyltransferases that are responsible for m^3^C20 of human cyto-tRNA^Ser^ and cyto-tRNA^Leu^ and m^3^C47:3 of human cyto-tRNA^eMet^ are still unknown.

Importantly, the methylation of m^3^C occurs on the Watson–Crick interface of cytosine and endows a positive charge on the nucleobase. This chemical character of m^3^C significantly alters its topological structure and base-paring capacity, which blocks the reverse transcription by high-fidelity reverse transcriptase or induces mismatch substitutions by low-fidelity reverse transcriptase during the cDNA synthesis process [100]. Moreover, the methylation of m^3^C32 by its modifiers requires a sequence context for the recognition of appropriate target tRNAs, such as human METTL2A/METTL2B methylate m^3^C32 of cyto-tRNA^Thr^ and cyto-tRNA^Arg^ in the presence of G35 and U36, respectively. Moreover, the modification state of A37 also plays a necessary function in the formation of m^3^C32 [101], suggesting a mutual regulation and crosstalk network for cellular tRNA modification control.

Functionally, increasing evidence shows the important role of tRNA m^3^C32 modification in regulating cellular translation rate and mitochondrial protein synthesis [98]. The deletion of METTL2A/METTL2B and METTL6 decreased the level of m^3^C32 modification on tRNA^Ser(GCT)^ and altered the translation of serine codon-biased mRNA [102]. Knockdown of a cofactor of METTL2, DALR anticodon binding domain containing 3 (DALRD3), leads to nearly complete loss of m^3^C32 on cyto-tRNA^Arg^, which might be associated with human developmental delay and epileptic encephalopathy [103].

Recently, METTL8 was found to methylate C32 to m^3^C32 for mt-tRNA^Thr^ and mt-tRNA^Ser^ [104,105,106] and be involved in regulating the maintenance of embryonic cortical neural stem cells in mice [107]. The modification of m^3^C32 promotes mitochondria tRNA folding and maintains tRNA structure stability, thereby enhancing the translation efficiency of mitochondrial proteins. Interestingly, the biological role of METTL8 is not restricted to mt-tRNA m^3^C32 modification; the generation of various METTL8 isoforms by alternative splicing diversifies the biological function of METTL8. As reported, some METTL8 isoforms could enter into mitochondria and serve as tRNA m^3^C32 modifiers, while some isoforms are retained in the nucleolus and function as a regulator in R loop formation [108].

#### 3.1.6. Pseudouridine (Ψ)

As the most prevalent modification, Ψ is present on various types of RNAs across three kingdoms [59,109]. On tRNA, Ψ is catalyzed by different Ψ synthases with specific tRNA base locations. Among these locations, Ψ55 is present in nearly all tRNAs at the position of the TΨC loop and plays pivotal roles in stabilizing tRNA tertiary structures and maintaining tRNA biological function in translation. In human tRNA, Ψ55 is catalyzed by nuclear TruB Pseudouridine Synthase Family Member 1 (TRUB1), mitochondrial TRUB2, and PUS10 [110]. Notably, PUS10 has two isoforms: one is cytoplasmic PUS10, which is responsible for the pseudouridinylation of Ψ54 and Ψ55; the other is the nuclear isoform of PUS10, which is catalytically inactive but has a specific binding affinity to tRNAs that contain unmodified U54/A54U55, thereby inhibiting nuclear TRUB1 activity on conversing U55 to Ψ55 [111]. The other positions of Ψ modifications on human tRNA are usually catalyzed by the PUS family (PUS1; PUS7) [110,112], which usually occurs on U1, U8, U26–28, U34, and U36.

As mentioned above, one important role of pseudouridinylation is to ensure the proper folding and stabilization of tRNA tertiary structures. The Watson–Crick base pairs of Ψ are the same as U, which is adenine A, but with greater thermodynamic stability than U-A pairs. For example, Ψ39 of tRNA^Lys^ was reported to increase the melting temperature by 5 °C, thus stabilizing tRNA from degradation. However, there is an exception whereby the Ψ8 modification that is catalyzed by PUS7 can reduce tRNA stability. After the knockout of PUS7 in human stem cells, although the overall amount of tRNA is unchanged, a reduction in 5′tsRNA from tRNA^Ala^, tRNA^Cys^, and tRNA^Val^ can be observed [113], while the overexpression of PUS7 enhances the biogenesis of 5′tsRNA^Ala/Cys^ and 5′tsRNA^Val^ with Ψ8 modification, which further sequesters the translation initiation factors and significantly represses the cellular translation rate.

#### 3.1.7. Queuosine (Q)

As a hyper-modified G analog, Q modification exists at the wobble position of tRNA^His^, tRNA^Asn^, tRNA^Tyr^, and tRNA^Asp^ [114] and directly participates in codon–anticodon recognition processes that contribute to translation fidelity and efficiency [73]. However, eukaryotes are unable to generate Q modification directly but depend on the source of queuine from diet and gut microbiota [115,116]. The Q modification on tRNAs with GUN anticodons is catalyzed by tRNA-G transglycosylase (TGT) in eukaryotes and bacteria [117,118]. The presence of Q can alter the structure of the DNMT2-tRNA complex, improve the transition state energy, and thereby stimulate DNMT2 activity, further enhancing the formation of m^5^C38 on tRNA^Asp^ [119,120]. Another aspect of the presence of Q modification is to protect full-length tRNAs from cleavage by angiogenin (ANG) and alter the tsRNA pools [114]. In fact, the most important effect is that Q-decoded codons can accelerate translation speeds, while the loss of Q modification reduces C38 methylation in tRNA^Asp(GUC)^, consequently leading to ribosomal stalling and the imbalanced translation of other codons. This effect is somehow sex-dependent, with female mice showing more severe impairments in learning and memory formation in the hippocampus [121]. The translation effect might lead to severe consequences such as endoplasmic reticulum stress and activation of the unfolded protein response [119].

**Table 1:** tRNA modifications and their associated pathologies.

tRNA Modification	Associated Pathology	References
m^5^C	Male germ cell differentiation Tumor metastasis Paternally acquired metabolic disorders	[57] [122] [52]
m^1^A	T cell proliferation post-antigen stimulation Mitochondrial disorders: MERRF (myoclonus epilepsy, ragged-red fibers)	[123] [124] [125]
m^1^G	Ehrlich ascites tumor; neuroblastoma Hereditary spastic paraparesis Neuropathy syndromes Combined oxidative phosphorylation deficiency 26 (COXPD26) Idiopathic non-cirrhotic portal hypertension; hepatopulmonary syndrome	[126] [78] [79] [80] [81]
m^7^G	Acute myeloid leukemia Bladder cancer Hepatocellular carcinoma Prostate cancer Stem cell fate determination Aging	[86,92] [93] [94] [95] [16] [97]
m^3^C	Human developmental delay and epileptic encephalopathy	[103]
Ψ	Mitochondrial disease	[127]
Q	Sex-dependent learning and memory formation in the hippocampus	[121]

### 3.2. Rebirth of tsRNA from tRNA

The generation of tsRNAs in cells primarily depends on the recognition and cleavage of tRNAs by endogenous nucleases (Figure 2b). These specific nucleases are capable of recognizing tRNAs based on their modification status and cleaving them at specific locations, producing tsRNA fragments with diverse lengths and sequences. Researchers found that most tsRNAs originate from mature tRNAs, but a small portion of tsRNAs are derived from precursor tRNAs. During the maturation process of precursor tRNAs (Pre-tRNAs), ribonuclease P (RNase P) can remove the 5′ end sequences of Pre-tRNAs to generate 5′-Leader-tsRNAs, while ribonuclease Z (RNase Z) and ElaC homolog protein 2 (ELAC2) can cleave the 3′ ends of Pre-tRNAs to produce 3′-Trailer-tsRNAs [128]. In the mammalian nuclease A family, ANG can cleave near the anticodon loop of mature tRNAs under stress conditions, producing 5′tsRNAs and 3′tsRNAs (also known as 5′tRNA halves and 3′tRNA halves) with lengths around 30–40 nucleotides. Ribonuclease Dicer can cleave near the D loop and TΨC loop to produce shorter 5′tsRNAs and 3′tsRNAs with lengths of around 18–25 nucleotides [129]. Additionally, RNase P can also target specific sequences (such as those GC-rich base pairs) in the stem of the tRNA anticodon loop for cleavage [130].

Studies have revealed a universal expression pattern of tsRNA in various tissues and cell types among animals and plants. Interestingly, the profiles of tsRNA show significant expression heterogeneity among different tissues, body fluids, and cancer samples. For instance, in terms of the total amount, tsRNAs are highly enriched in serum and mature sperm, in which serum tsRNAs are dominantly encapsulated by the serum protein complex and sensitive to body active infection and inflammation, while sperm tsRNA exhibits enrichment in the sperm head [131,132] and sensitively responds to environmental exposures, nutrition alteration, mental trauma, and aging. Moreover, besides serum and sperm, growing evidence shows considerable tsRNA abundance in various cancers with distinct signatures, suggesting a promising application direction of tsRNAs as diagnostic biomarkers of cancers. Moreover, with the development of small RNA high-throughput sequencing techniques, such as panoramic RNA display by overcoming RNA modification aborted sequencing (PANDORA-seq) and others [133,134,135]^,^ researchers can precisely characterize and detect the noncanonical small RNAs, such as tsRNA and rRNA-derived small (rsRNA), across tissues and cells under physiological and pathological conditions, which facilitates the identification of functional small non-coding RNA (sncRNA) in biological processes of inflammation, aging, stem cell fate determination, disease, etc.

## 4. Revisit the Regulatory Mechanisms of tRNA and tsRNA

### 4.1. tRNA: Focus on Both Main and Side Businesses

The indispensable role of tRNA in decoding mRNA and facilitating protein synthesis is undeniable. Alterations in tRNA pools, specific tRNA species, RNA modifications, and charging levels can significantly impact protein synthesis efficiency. These effects may arise from variations in tRNA availability or by influencing critical processes like codon–anticodon pairing [7,8,37]. While the primary function of tRNA is mRNA decoding during translation, emerging evidence from DNA/RNA sequence complementarity rules and structural biology suggests that tRNAs also possess functions beyond translation. This expanding understanding of tRNA mechanisms provides valuable insights into their broader biological roles.

#### 4.1.1. Being Primers for Reverse Transcription Transposons

Transposable elements (TEs) play a significant role in genome evolution, with reverse transcription transposons representing a crucial category. These elements can utilize RNA as an intermediary, reverse transcribe it into DNA, and subsequently integrate it into the host genome [136]. Reverse transcription transposons are primarily divided into two categories: Long terminal repeat-retrotransposons (LTRs; also known as Endogenous Retroviruses, ERVs) and non-LTR reverse transcription transposons (e.g., long interspersed nuclear elements (LINEs), short interspersed nuclear elements (SINEs)) [136]. Research indicates that the 3′ end of mature tRNAs can serve as a primer for LTR reverse transcription, anchoring to the highly conserved primer binding sequence (PBS), thereby initiating the reverse transcription process [137]. This mechanism has been observed in a range of retroviruses, including human immunodeficiency virus (HIV), human T cell leukemia virus type 1, Rous sarcoma virus, Moloney murine leukemia virus, and Mouse mammary tumor virus [129]. Further exploration of this relationship could provide deeper insights into both the mechanisms of viral replication and the broader implications for host genome evolution.

#### 4.1.2. Binding to DNA/RNA

tRNA can regulate gene expression by binding to DNA through a sequence base-pairing strategy. During the development of zebrafish embryos, full-length tRNA can form a stable tRNA:DNA hybrid at the tRNA gene loci, thus hindering the transcription process of tRNA genes [23]. However, the 5′tsRNAs, such as 5′tsRNA^Gly(GCC)^ and 5′tsRNA^Glu(CTC)^, could compete with full-length tRNA to bind with the template strand of tRNA genes, which forms an unstable 5′tsRNA:DNA hybrid compared to the tRNA:DNA hybrid, thus facilitating the transcription of tRNA genes [23]. In addition, Bacteriophage could utilize *E. coli* tRNA as a primer to initiate lagging strand DNA synthesis [138]. It has been observed that the growth defect that is caused by the absence of DNA primase in Bacteriophage can be rescued by mutations in T7 gene 5.5 [138]. These mutations on gene 5.5 directly reduce the abundance of gp5.5, which hinders the formation of the gp5.5-tRNA-H-NS complex [138]. As a result, the additional free tRNA can function as a substitute primer for Bacteriophage DNA replication [138]. This strategy allows Bacteriophage to flexibly use host tRNAs for survival and amplification [138]. In addition to the above-mentioned role of tRNA as a retrotransposon primer, tRNA can also interact with mRNA to fulfill functions. For example, research has demonstrated that initiator-tRNA^Met^ (tRNA^iMet^) can regulate pre-mRNA splicing through base-pairing with the start codon of targeted mRNA, which is independent of its aminoacylation status [139]. Additionally, another study found that uncharged tRNA^Asp^ can adopt an atypical structure to interact with Alu elements in the 3′ Untranslated Region (3′UTR), influencing the selection of polyadenylation sites [140]. These functions of tRNA, involving sequence pairing with DNA/RNA or conformational changes, shift the focus away from the anticodon sequence, emphasizing the functional significance of the entire tRNA sequence and the secondary or tertiary structures.

#### 4.1.3. Interacting with Proteins

Research has shown that tRNA can also bind to specific proteins, thereby influencing the function of proteins and exerting an effect. For instance, tRNA has been identified as a starvation sensor. In prokaryotes, deacylated tRNAs accumulate under stress conditions, and RelA interacts with these uncharged tRNAs in the vacant ribosomal A-site, triggering the synthesis of (p)ppGpp, a critical signaling molecule that initiates the bacterial stringent response [141]. In eukaryotes, deacylated tRNAs bind to the protein kinase general control nonderepressible 2 (GCN2), which downregulates translation and activates its downstream functions [142]. In addition, the binding of mitochondrial and cytoplasmic tRNA to cytochrome c prevents the interaction between cytochrome c and Apaf-1, blocking Apaf-1 oligomerization and caspase activation, thereby inhibiting cell apoptosis [143]. Additionally, research has indicated that the capsid binding membrane protein pp150 on human cytomegalovirus (HCMV) binds to tRNA, and one proposed biological function of this binding is to enhance the stability of the capsid protein, allowing the virus to withstand its massive genome [144]. Specifically, several studies have highlighted the role of tRNA in immune regulation. For instance, research has shown that tRNA can interact with interferon-stimulated gene (ISG) proteins (e.g., Schlafen11(SLFN11), SLFN13, interferon 5 (IFIT5)), potentially regulating protein translation [145,146,147]. Additionally, the Gm18 modification of tRNA can inhibit the activation of 7 toll-like receptor 7 (TLR), which may represent a strategy employed by bacteria to weaken host defense responses through tRNA modification [148]. These protein–tRNA interactions are crucial for regulating cellular responses to stress, apoptosis, and immune challenges, showing that tRNA’s role in the cell extends far beyond translation. Understanding these interactions provides deeper insight into the complex regulatory networks within cells, highlighting the multifaceted roles of tRNA in modulating protein function and influencing key cellular processes.

### 4.2. tsRNA Regulation: Structure Dictates Function

Given the heterogeneous nature of tsRNAs, which exhibit variations in origin, sequence, length, and modifications, their regulatory mechanisms are diverse and encompass a wide range of functions. These regulatory functions of tsRNAs have been elucidated and classified according to the various stages of cellular activity, including transcription, post-transcriptional regulation, and translation control. It is noteworthy that tsRNAs can form higher-order structures stabilized by intra or intermolecular interactions that are influenced by intrinsic sequence pairing propensity or specific RNA modifications. This structural versatility contributes to a more complex and diverse regulatory landscape of tsRNAs, exerting regulatory effects distinct from those mediated by sequence complementarity.

#### 4.2.1. Sequence Complementarity

As they are generated from tRNAs, tsRNAs share a similar sequence with their tRNA precursors, which enables tsRNAs to compete with tRNA on the regulatory occasions where a sequence complementary mechanism is needed. For example, 5′tsRNA^Gly(GCC)^ can compete with tRNA^Gly(GCC)^ to base-pair with tDNA during the embryonic development of zebrafish, thus inhibiting the formation of the tRNA-dependent stable transcription-inhibiting R loop structure (a three-stranded structure composed of one RNA strand and two DNA strands) around tRNA genes, which ultimately promotes the transcription of tDNAs. On another occasion, an 18 nt 3′tsRNA^Lys(AAA)^ could compete with mature tRNA^Lys(AAA)^ to occupy the PBS in a complete base-pairing manner, leading to reverse transcription blocking and impeding the cDNA synthesis process of long terminal repeat retrotransposon, which utilizes tRNA^Lys(AAA)^ as a reverse transcription primer [149]. Interestingly, a 22 nt 3′tsRNA ^Lys(AAA)^ could effectively silence ERV in a microRNA -like manner with a 2 bp mismatch when binding with the PBS of ERV. Therefore, 3′tsRNAs ^Lys(AAA)^ with different nucleotide lengths could suppress the LTR-retrotransposon activity both through impeding the cDNA synthesis of ERV and silencing ERV transcripts to protect the cells during reprogramming [149].

The direct sequence complementary binding mechanism of tsRNA could also occur between tsRNA and mRNA in a protein-dependent or independent manner. It was reported that 3′tsRNA^Leu(CAG)^ can complementary base-pair with the structured region of ribosome proteins’ transcripts, such as Ribosomal proteins 28 (RPS28) and RPS15. The base-pairing of 3′tsRNA^Leu(CAG)^ with mRNA unfolds the intramolecular structure of mRNA to allow ribosomes to pass through the structured mRNAs smoothly, thereby promoting the translation elongation process of ribosome proteins and the overall cellular translation process [150]. In addition to the sequence-complementary pairing that occurs between tsRNA and mRNA coding sequences, some tsRNAs could also bind to the intron sequences of pre-mRNAs, which leads to the silencing of nascent RNA of the target gene (nascent RNA silencing) [151].

In consideration of the protein-dependent manner, some tsRNAs can operate a miRNA-like mechanism to bind to Argonaute (AGO) proteins to form an RNA-induced silencing complex (RISC), leading to the promotion of mRNA degradation or the inhibition of mRNA translation. This interaction typically relies on precise nucleotide pairing between tsRNA and the target mRNA. One occasion is that tsRNAs target mRNA 3′UTR with AGO proteins in a sequence-complementary manner and lead to the degradation of target mRNAs [152,153]. Moreover, the regulatory effects of tsRNAs based on sequence complementarity sometimes only require a few nucleotide base-pairs. In fruit flies, tsRNAs target the conservative regions of mRNA through short seed sequences consisting of seven nucleotides, without affecting the mRNA stability, but inhibit the translation of target gene mRNA by AGO proteins [154]. Interestingly, these tsRNAs showed a tendency to target critical genes within the translation complex, such as ribosome proteins, which could expand the translation inhibition from several transcripts to raise an overall translation inhibition wave in cells.

#### 4.2.2. tsRNA Secondary Structure

In addition to a sequence-dependent regulatory mechanism, tsRNAs can also function independently of the sequence complementary principle but through its unique secondary or three-dimensional structures [155]. The spatial structure of some tsRNAs may present challenges during the small RNA sequencing process, potentially leading to the under-discovery of genuinely existing tsRNAs. Moreover, due to the highly modified characteristics of tsRNA, the investigation of tsRNA by using synthesized unmodified tsRNA mimics could not fully elucidate their sequence-independent functions, thereby impeding our understanding of the regulatory mechanisms of tsRNAs. However, growing evidence suggests that many tsRNAs indeed possess diverse secondary structures based on their sequence composition or various RNA modifications, and the regulatory function triggered by spatial interactions with other molecules should not be underestimated. On many occasions, the spatial conformation and chemical RNA modification signatures of tsRNAs contribute to the recognition and binding affinity by RNA binding proteins. For example, the ribonucleoprotein (RNP) of *Tetrahymena* consists of spatially structured tsRNAs that bind to AGO/PIWI in an aptamer-like manner to promote the processing of nuclear rRNA [156,157]. In mammalian cells, 5′tsRNAs containing a terminal oligo-G motif (TOG), such as 5′tsRNA^Ala^ and 5′tsRNA^Cys^ (~30 nt), can fold into intermolecular RNA G-quadruplex (RNA G4) structures. The tsRNAs with RNA G4 structures can either competitively bind to the translation initiation complex eIF4G/eIF4E or bind to Y-Box Binding Protein 1 (YBX1) to prompt mRNA packaging into stress granules, thereby hindering translation initiation and resulting in global translation repression. Moreover, 5′tsRNA^Ala/Cys^ possesses a Ψ modification at the position of Ψ8, which can also displace m^7^G-capped mRNA from polyadenylate binding protein 1 (PABPC1) and eukaryotic translation initiation factor 4A/G (eIF4A/G) to abolish the formation of the translation initiation complex, while tsRNA with the same sequence but without the Ψ8 modification cannot [158]. However, tsRNA with distinct sequences and secondary structures can be recognized by the same RNA binding proteins under different circumstances. As mentioned above, Y-box binding protein 1 (YBX1) can bind with tsRNA with intermolecular RNA G4 to facilitate the assembly of stress granules in regulating overall cellular translation. Evidence also shows that YBX1 can recognize and bind to inner’tsRNA derived from the anticodon loop of tRNA^Gly(TCC)^, tRNA^Glu(YTC)^, tRNA^Asp(GTC)^, etc., with the SCUBYC motif under hypoxia stress [159]. These hypoxia stress-induced tsRNAs competitively bind to YBX1 by displacing mRNA, resulting in the destabilization of pro-oncogenic transcripts and the repression of tumor metastasis under hypoxia. However, the mechanism of tsRNA-YBX1 recognition and binding still needs further investigation.

## 5. Regulators in Cell Fate Determination and Embryonic Development

The differentiation of stem cells into various cell types is modulated by gene temporal and spatial transcription and translation and determined by cell-specific proteomics. During this process, the composition and abundance of the tRNA pools are adjusted to meet the translational demand of cells and are thereby involved in cell fate determination. Evidence has shown that genes associated with proliferation and differentiation exhibit distinct codon usage patterns. Correspondingly, the tRNA pools in cells show different profiles during proliferation and differentiation, reinforcing either state [160]. For instance, during the differentiation of human induced pluripotent stem cells (hiPSCs), the tRNA transcript pool undergoes extensive remodeling [34]. Moreover, distinct subsets of the tRNA pools were observed under different cell states to respond to biological activities in human cells [32], among which, some tRNAs were defined as “proliferation-tRNAs”, while some tRNA populations were defined as “differentiation-tRNAs”, according to their expression signatures in distinct cell types. Noa Aharon-Hefetz et al. found that the “proliferation-tRNAs” were upregulated during normal and cancerous cell division, whereas “differentiation-tRNAs” were more active in non-dividing but differentiated cells, suggesting that tRNA plays a crucial role in regulating cell fate determination. Importantly, the heterogeneity of tRNA in cells is precisely regulated to maintain cell-specific tRNA pools and fulfill the translation demand during cell fate determination [161]. During hiPSC differentiation, the chromatin state of tRNA genes modulated by histone modifications, such as H3K4me3 and H3K27me3, determines the recruiting of RNA pol III on specific tRNA genes, thus controlling tRNA isodecoder transcripts in cellular tRNA pools. However, the RNA pol III repressor MAF1 could stabilize cellular tRNA isotype pools by regulating the activation of RNA pol III through the mechanistic target of the rapamycin kinase (mTOR) signal pathway. This stabilization minimizes the risk of ribosomal errors and protein misfolding due to fluctuations in decoding rates [34].

During embryonic development, the expression profiles of tRNAs and tsRNAs show dynamic regulation. After fertilization and zygotic genome activation (ZGA), the transcription activity is largely enhanced to support the developmental process, triggering extensive reprogramming and a global increase in translation activity. At the same time, the repertoire of tRNA undergoes necessary alterations to adapt to the translation process during embryonic development. For example, during the embryonic development of zebrafish, the repertoire of tRNA isodecoder and tRNA modification signatures undergoes substantial alteration around the onset of gastrulation [162]. Repression of the translation of tRNA^Gly(GCC)^ and tRNA^Glu(CTC)^ in zebrafish embryos leads to early embryonic lethality [23]. Similarly, the deletion of tRNA^Phe^ isodecoder genes impairs the embryonic development of mice, in which the isodecoder tRNA^Phe^1-1, required for neuronal function, shows the most serious lethal effect [17]. This evidence suggests a dispensable role of proper tRNA pools in maintaining early embryonic development.

In addition to the modulation of tRNA abundance, the regulation of tRNA modification levels also plays pivotal roles in controlling cellular translation efficiency in stem cells. For instance, the depletion of the Ψ “writer” PUS7 significantly impairs mesoderm specification in hESCs [113]. Similarly, the knockout of the m^7^G methyltransferase *Mettl1* promotes differentiation towards the mesoderm and endoderm while impairing differentiation towards the neural lineage in mESCs [16]. Moreover, a study showed that pluripotent cells exhibit distinct codon usage signatures, with a preference for translation by inosine-modified tRNAs in self-renewing hESCs. tRNA adenosine deaminase ADAT2-ADAT3, which catalyzes the conversion of adenosine to inosine at position 34 (A-to-I) of certain tRNAs, shows higher levels in self-renewing hESCs, as well as a higher abundance of I34-modified tRNAs, while the differentiation treatment of hESCs leads to the downregulation of ADAT2-ADAT3 and I34 tRNA abundance. Additionally, growing evidence shows the crucial roles of tRNA modification enzymes in maintaining normal lineage specification during early embryogenesis. The deletion of *NSun3*, a tRNA m^5^C modifier, leads to embryonic lethality between E10.5 and E12.5 in mice [163], and the deletion of elastin-like polypeptide 3 (ELP3), a tRNA U methylation enzyme, causes early embryonic lethality between E7.5 and E12.5 with severe growth retardation in mice [164].

Instead of the precise regulation of tRNA pools, the expression profiles of tsRNAs show dynamic alteration during embryonic development and stem cell fate commitment. tsRNAs derived from tRNA^Gly^, tRNA^Glu^, and tRNA^Val^ show significant upregulation after fertilization and become particularly abundant in the eight-cell stage embryo in mice [165]. Notably, the inhibition of tsRNA^Glu(CTC)^ in an in vitro conditioned medium of preimplantation embryos results in the heightened expression of actin-related genes, fostering the formation of actin-based trophectoderm projections and significantly increasing embryo hatching rates [166]. In embryoid body (EB) formation assays, the upregulation of tsRNAs derived from tRNA^Ala^, tRNA^Arg^, tRNA^Glu^, tRNA^His^, and tRNA^Lys^ is observed during mESC differentiation. Transfecting these tsRNAs into mESCs enhances lineage differentiation during EB formation, suggesting that certain tsRNAs are directly involved in the differentiation process of mESCs [133]. In differentiating mESCs, the set of 5′tsRNAs remains consistently enriched. The use of antisense oligonucleotides to inhibit the upregulation of 5′tsRNAs suppresses the differentiation process in mESCs, maintaining their pluripotency. This finding underscores the role of 5′tsRNAs in promoting differentiation while inhibiting self-renewal [167].

## 6. tsRNA: From Sensor of Environment to Epigenetic Inheritance Messenger

**Figure 3:**
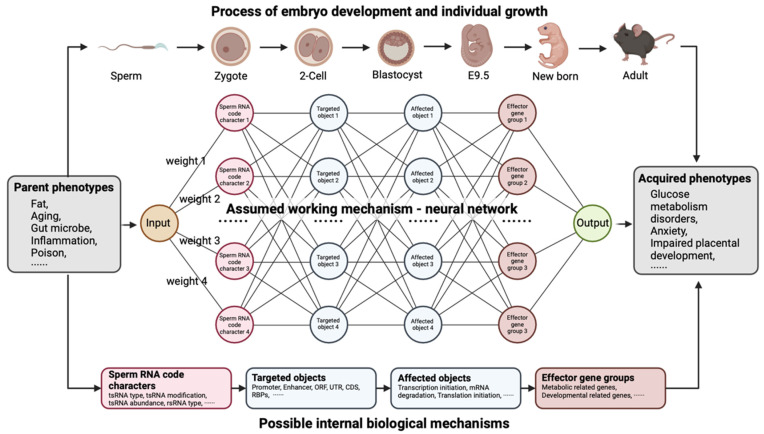
Neural network model for transmitting parental traits to offspring through the sperm RNA code. The paternally acquired phenotypes are encoded as the sperm RNA code, with various features. It is possible that these features may interact with each other, either directly or indirectly, to influence the biological process in the early embryo and subsequently affect the phenotypes of the offspring. Created in BioRender. Yang, Y. (2024) BioRender.com/x33h223 (accessed on 15 August 2024).

SncRNA profiles (including piRNAs, miRNAs, tsRNAs, rsRNAs, etc.) [133] in male germ cells undergo dramatic remodeling during spermatogenesis in testis and sperm maturation in the epididymis, resulting in a significant special sncRNA profile in mature sperm [168]. The literature shows that the expression and RNA modification profiles of sperm sncRNAs are highly sensitive to environmental perturbations. Environment exposures, such as nutritional alteration stress (high-fat diet or low-protein diet), fetal trauma, chemical toxicants, and aging are capable of shaping sperm sncRNA signatures (including sncRNA expression and RNA modification profiles), thus generating the “sperm RNA code” that encodes paternal life experience [169] and further leading to the intergenerational or transgenerational epigenetic inheritance of paternally acquired phenotypes in the offspring [170]. As we propose here, the neural network model of sperm RNA code-mediated paternal intergenerational inheritance is a possible mechanistic model that may help deepen our understanding of sperm RNA-mediated epigenetic inheritance (Figure 3). Under environmental stress, the paternal exposures act as information inputs that are parsed into various sperm RNA signatures, such as tsRNA features, to form a specific “sperm RNA code”. During fertilization, sperm RNA enters the oocyte along with sperm and targets specific regulatory elements, such as promoters, enhancers, open reading frames (ORFs), UTRs, coding sequences (CDS), and RNA-binding proteins (RBPs) through diverse mechanisms [24] during early embryonic development. The regulatory effects further influence embryonic epigenetic reprogramming and transcriptional and translational activities [25], subsequently affecting specific biological processes and ultimately impacting embryonic development. These acquired traits gradually manifest, completing the process of phenotypic intergenerational inheritance during the development and growth of offspring.

As a crucial part of the “sperm RNA code”, tsRNA is one of the most abundant sncRNAs with an apparent nucleus and mitochondrial localization in sperm. tsRNA is characterized by various RNA modifications and diverse regulatory mechanisms in biological processes, expanding the information coding capacity of tsRNAs during fertilization and embryonic development [133]. Growing evidence confirms that sperm tsRNAs are capable of responding to various environmental stresses, such as nutritional changes, age factors, hypoxic conditions, inflammation, gut microbiota, and chemical toxicity in mammals [132,169,171,172,173,174,175]. In humans, one week of a high-sugar diet is sufficient to alter the expression signature of tsRNA and rsRNA in sperm [176], and the alteration of the sperm RNA code after 6 months of high-fat-diet treatment is capable of independently transmitting the paternal acquired phenotype to the offspring, resulting in metabolic disorders in the progeny [169]. Moreover, aging-induced alterations of sperm tsRNA profiles have been shown to induce anxiety-like behaviors in the offspring [172]. Gut microbiota dysbiosis-induced upregulation of tsRNA^Gly(GCC)^ in the sperm increases the risk of impaired placental development and premature death in mice offspring [175]. Additionally, RNA modifications also serve as key components of the sperm RNA code in response to environmental stimuli. We previously found that global modifications in mouse testes and mature sperm underwent significant remodeling under hypoxic conditions [177], while m^2^G and m^5^C modifications notably increased on 30–40 nt sperm sncRNAs after 6 months of high-fat-diet treatment [169]. However, the deletion of tRNA m^5^C38 methyltransferase enzyme *Dnmt2* repressed the upregulation of m^5^C and m^2^G modification abundance under high-fat-diet treatment. Consequently, neither the total RNA nor the tsRNAs in sperm could transmit the acquired metabolic disorder phenotype to the offspring [52]. These results suggest that RNA modifications and their modifiers may serve as key regulators in sperm sncRNA-mediated intergenerational epigenetic inheritance.

## 7. Emerging Stars in Disease and Therapeutic Modulation

A growing body of evidence has established a significant link between tRNA biology and a range of pathological conditions, including neurodegenerative diseases [178], metabolic disorders [179], and cancer [180], among others. While some diseases involve mutations on tRNA genes, particularly mitochondrial tRNA mutations, which have been extensively summarized in several excellent reviews [41,181], an increasing number of studies have identified the etiology of some diseases, with alterations in RNA pol III, transcription factors, tRNA gene methylation, histone methylation, tRNA modifying enzymes, and aminoacyl–tRNA synthetases [41,182,183]. These factors collectively modulate the landscape of cellular tRNA expression profiles, tRNA modification signatures, and amino acid charging levels, thereby dysregulating tRNA biological functions and contributing to disease pathogenesis. A prominent example of how altered tRNA expression profiles affect biological functions is found in cancer, where the composition of tRNA pools favors the translation of oncogenic factors rich in specific codons, promoting disease progression [27]. Furthermore, modifications in the tRNA epitranscriptome impact anticodon–codon affinity, the recognition of wobble base positions, and tRNA stability [7,8,9], all of which influence translation efficiency. For instance, the loss of PUS1 function leads to the absence of Ψ modifications at specific sites on tRNA (positions 27 and 28), which is associated with the development of mitochondrial myopathy and sideroblastic anemia (MLASA) [179]. It is hypothesized that these modifications affect tRNA structure formation and stability. Additionally, tRNA charging levels play a crucial role in determining the availability of tRNAs for translation. In the mitochondrial encephalomyopathy with lactic acidosis and stroke-like episodes (MELAS) syndrome cell model, it was observed that the content of mt-tRNA^Leu(UUR)^ decreased by more than 50% compared to normal cells, and its aminoacylation efficiency was similarly reduced by 50% [184]. When considering both the reduction in mt-tRNA^Leu(UUR)^ levels and the aminoacylation efficiency, the actual level of aminoacylated mt-tRNA^Leu(UUR)^ was estimated to be only 25% to 30% of that in the control group [184], emphasizing the association of abnormal aminoacylation levels with disease. Instead of tRNA, tsRNAs are emerging as novel diagnostic biomarkers and crucial regulators in various diseases. The diversity of expression signatures of tsRNAs in human diseases such as epilepsy [185], acute myeloid leukemia [28], chronic lymphocytic leukemia [186], lung cancer [187], breast cancer [188], and aging [189] enable tsRNAs to serve as promising candidates for diagnostic and therapy applications. Moreover, a recent study found a significant production of tsRNA^Glu(CTC)^ within mitochondria in brains with Alzheimer’s disease. The accumulation of tsRNA^Glu(CTC)^ competes with mitochondrial leucine tRNA for binding to leucyl–tRNA synthetase 2 (LaRs2), disrupting the aminoacylation of leucine tRNA and impairing the translation of mitochondrial proteins. This disruption affects both the cristae structure and glutamate synthesis, contributing to accelerated brain aging and the progression of Alzheimer’s disease [189].

The clinical application of tRNA in therapeutic strategies for certain diseases has proved to be quite exciting, particularly with the use of suppressor tRNA (Sup-tRNA). Sup-tRNA is a unique type of tRNA capable of recognizing and decoding stop codons (such as UGA, UAA, and UAG) on mRNA, allowing for the insertion of a specific amino acid during protein synthesis rather than terminating translation [190]. This capability makes Sup-tRNA a potentially powerful tool in both basic research and clinical treatment, especially in solving genetic disorders where premature stop codons (nonsense mutations) cause the early termination of protein synthesis, leading to functional deficiencies. Initially, researchers employed natural sup-tRNA^Arg^ to restore the expression of E-cadherin from mutated CDH1 genes to retard disease progression in patients with hereditary diffuse gastric cancer (HDGC) [191]. By individually fine-tuning sequences to fit the physicochemical properties of the amino acids they carried, natural tRNAs could also be altered to be an effective sup-tRNA that could fully bind alanine on the stop codon UGA, capable of restoring the production of the nonsense mutated functional protein in mice [29]. The researchers then screened Sup-tRNA using high-throughput assays to select the sequences best suited for in vitro and in vivo nonsense suppression. John D. Lueck and others engineered inhibitory tRNA, known as anticodon-engineered transfer RNAs (ACE-tRNA), to bind to endogenous aminoacyl–tRNA synthetases and allow the corresponding amino acid to bind, forming aminoacyl–tRNA. They then introduced the normal protein’s corresponding amino acid at the PTC site, forming a sequence and full-length accurate normal protein [192]. In addition, a groundbreaking study demonstrated the potential of Sup-tRNA in treating genetic diseases caused by nonsense mutations. Delivering Sup-tRNA via the adeno-associated virus (AAV) in mice effectively overcame these genetic mutations [30]. This innovative gene therapy approach utilizes Sup-tRNA to read through premature stop codons, thereby restoring the production of intact and functional proteins under endogenous gene regulation. Research indicates that this method is both safe and effective, with therapeutic benefits lasting over six months after a single treatment, without disrupting global protein synthesis or tRNA homeostasis [30]. Additionally, mutations in aminoacyl–tRNA synthetases have been implicated in Charcot–Marie–Tooth (CMT, a genetic neuropathy with muscle weakness and atrophy in the lower limbs), causing abnormal tRNA binding and impaired release. This disruption leads to a deficiency of tRNA in cells, which subsequently disrupts protein synthesis [193]. However, studies have shown that overexpressing tRNA in CMT disease models in fruit flies and mice can restore protein synthesis, alleviate neuropathic symptoms, and reduce cellular stress responses [193]. This research not only reveals a potential molecular mechanism underlying CMT disease but also suggests that increasing tRNA levels may hold therapeutic promise for this condition.

These emerging insights into tRNA and tsRNA biology highlight their crucial roles both in regulating the pathogenesis of various diseases and in serving as potential diagnostic biomarkers and therapeutic targets. The intricate involvement of tRNA in protein synthesis and disease mechanisms, alongside the versatile regulatory functions of tsRNAs, reflects a growing understanding of RNA’s broader impact on human health. These small RNAs could become stars in novel treatment strategies, offering precision medicine approaches that leverage their unique properties and interactions within cellular processes.

## 8. Conclusions and Perspectives

The diversity and heterogeneity of tRNA have led to a significant advancement in our understanding of the tRNA world. In fact, the organisms have evolved multiple redundancies of tRNA genes to ensure the reliable functioning of tRNAs. The number of tRNA genes varies among different species, with mice, humans, and other primates having between 400 and 700 tRNA genes, while zebrafish have approximately 10,000 [194]. In addition to the considerable diversity of tRNA gene copy numbers, the cellular tRNA pool is also intricately regulated. On the transcription level, the gene location of specific tRNAs, chromatin accessibility, activity of the Pol III complex, and selective binding of transcription factors play pivotal roles in regulating the transcription initiation of tRNA genes [17,36,195]. After transcription, the maturation of tRNAs is also tightly controlled by a complex network that consists of RNAases, CCA tailoring processes, RNA modification enzymes, and aminoacyl–tRNA synthetases [196]. These regulatory processes exert influences over the composition of the tRNA isoacceptors and isodecoders within the cell, forming cell-specific tRNA pools [35,161]. For instance, external alterations could influence tRNA total abundance, while specific tRNA isoacceptor abundance could regulate translation rates to meet tissue-specific translation needs. Additionally, tRNA modifications also play an important role in the formation and maintenance of the tRNA cloverleaf-like secondary structure and L-shaped tertiary structure, while some tRNA modifications are subjected to intricate regulation during translation, which governs the fidelity and efficiency of codon–anticodon recognition, conveying information beyond the tRNA sequence [197,198,199].

Furthermore, the non-classical functions of tRNAs encompass the regulation of transposon activity, modulation of tRNA gene expression, and participation in cellular immune regulation and viral replication. These observed biological functions illustrate the complex regulatory roles of tRNAs. It is noteworthy that tRNAs are also capable of generating tsRNAs by fragmentation in response to cellular requirements [26]. This process allows tRNAs to participate in biological regulation in the form of a new regulatory non-coding small RNA. As expected, tsRNAs exhibit multifaceted regulatory effects at the transcriptional, post-transcriptional, and translational levels through diverse mechanisms [200]. This regulatory strategy is highly sophisticated, whereby intact tRNAs engage in regulatory functions, both classical and non-classical, and, when necessary, generate new sncRNAs to meet the regulatory demands of the cell, thus enabling an efficient and multifaceted mode of regulation.

In recent years, the establishment of new high-throughput technologies for detecting tRNA and tsRNA profiles has provided us with a glimpse into the tRNA and tsRNA world during various physiological and pathological processes, unveiling the potential application of tRNA and tsRNA as diagnostic biomarkers and therapeutic strategies for various diseases [182,201,202]. Studies have already demonstrated that the manipulation of tRNAs can be used to treat genetic diseases [193,203]. Similarly, as a novel class of small RNAs, tsRNAs show great potential for application in disease diagnosis. However, several methodological challenges remain on the path to practical applications. Firstly, how can we achieve highly accurate high-throughput detection of every type of modification on tRNA? Due to the complexity and diversity of tRNA, current technologies sometimes struggle to accurately distinguish between modification types or sites. In the future, analyses of tRNA heterogeneity and tsRNA functions, much like mRNA research, are expected to become a routine part of multi-omics studies. A deeper and more detailed understanding of the tRNA landscape may allow us to gain a “God’s eye view,” allowing for the early prediction of cell fate or disease progression. Taken together, this lays the foundation for potential therapies targeting tRNA modifications and tsRNA pathways, as well as early diagnostic biomarkers.

## Abbreviations

Nucleotides: A—Adenine; U—Uracil; C—Cytosine; G—Guanine. Amino Acids: Ala—Alanine; Arg—Arginine; Asn—Asparagine; Asp—Aspartic acid; Cys—Cysteine; Glu—Glutamic acid; Gln—Glutamine; Gly—Glycine; His—Histidine; Ile—Isoleucine; Leu—Leucine; Lys—Lysine; Met—Methionine; Phe—Phenylalanine; Pro—Proline; Ser—Serine; Thr—Threonine; Trp—Tryptophan; Tyr—Tyrosine; Val—Valine.
